# Cerebrospinal fluid shunt–associated surgical site infection with three-month versus twelve-month surveillance periods in Canadian hospitals

**DOI:** 10.1017/ice.2022.119

**Published:** 2023-06

**Authors:** Kelly B. Choi, Vivienne Steele, John Conly, Blanda Chow, Jeannette L. Comeau, Joanne Embree, Bonita E. Lee, Marie-Astrid Lefebvre, Robyn Mitchell, Linda Pelude, Allyson L. Shephard, Joanne M. Langley, for the Canadian Nosocomial Infection Surveillance Program (CNISP)

**Affiliations:** 1 Public Health Agency of Canada, Ottawa, Ontario, Canada; 2 Alberta Health Services, Calgary, Alberta, Canada; 3 University of Calgary, Alberta, Canada; 4 Canadian Center for Vaccinology (Dalhousie University, IWK Health and Nova Scotia Health), Halifax, Nova Scotia, Canada; 5 Dalhousie University, Halifax, Nova Scotia, Canada; 6 University of Manitoba, Winnipeg, Manitoba, Canada; 7 Shared Health Manitoba, Winnipeg, Manitoba, Canada; 8 Stollery Children’s hospital, Edmonton, Alberta; 9 Montreal Children’s Hospital, McGill University Health Centre, Montreal, Québec, Canada; 10 Children’s Hospital of Eastern Ontario, Ottawa, Ontario, Canada

## Abstract

Cerebrospinal fluid shunt–associated surgical site infection surveillance for 3 months compared to 12 months after surgery captures 83% of cases with no significant differences in patient characteristics, surgery types, or pathogens. A shorter 3-month follow-up can reduce resource use and allow for more timely reporting of healthcare-associated infection rates for hospitals.

Cerebrospinal fluid (CSF) shunt surgeries provide lifesaving treatment for hydrocephalus, but they are frequently associated with revision surgeries, infections, and substantial clinical and financial consequences.^1^ Postoperative shunt infections are the most costly implant-related infection to treat in the United States,^2^ requiring hospital readmission with potential surgery and critical care.^3^ Despite advances in perioperative care and shunt technology, rates of complication and revision surgery remain relatively high,^4^ highlighting the need for efforts to prevent infections.

CSF shunt–associated surgical site infections (CSF shunt SSIs) are a target for many infection prevention and control (IPAC) surveillance programs. Although most CSF shunt SSIs occur within a few months of surgery,^5^ late infections also occur.^3^ In 2013, the Centers for Disease Control and Prevention (CDC) National Healthcare Safety Network (NHSN) shortened the follow-up period for identification of an SSI from 12 months to 3 months after surgery.^6^ Other national systems have also reduced the surveillance follow-up period for surgery-associated healthcare-associated infection (HAI) from 12 months to 3 months^7^; however, no consensus on follow-up time has been reached, and follow-up periods vary globally.

The Canadian Nosocomial Infection Surveillance program (CNISP), a collaboration between the Public Health Agency of Canada (PHAC), the Association of Medical Microbiology and Infectious Disease (AMMI) Canada, and sentinel hospitals across Canada conducts surveillance on HAIs and provides these data to hospital IPAC programs. The CNISP has conducted surveillance on CSF shunt SSIs since 2000 and has reported rates based on a 12-month postsurgery surveillance period.^5^ Here, we have compared 3-month versus 12-month follow-up to determine the nature of case ascertainment for the 2 periods.

## Methods

Patients of any age who underwent placement of a new internalized CSF shunt, or revision of an existing internalized shunt between January 1, 2009, and December 31, 2018, in a CNISP-participating hospital were eligible. Patients were followed for 12 months after surgery for the primary outcome of a CSF shunt SSI by review of laboratory and health records. Patients with externalized shunting devices or whose CSF was culture positive at the time of surgery were excluded (Supplementary Table 1 online). Trained infection control professionals conducted chart reviews using a standardized case report form. A CSF shunt SSI was defined as the identification of a bacterial or fungal pathogen(s) from the CSF associated with at least 1 of the following: fever or signs or symptoms of neurological or abdominal illness or of shunt malfunction or obstruction (Supplementary Table 1 online). Data were submitted to the PHAC for data cleaning and analyses.

Annual incidence rates at 3 and 12 months were calculated as the number of CSF shunt SSI cases per 100 shunt surgeries. Rates were calculated by age group (adult ≥18 years vs pediatric <18 years), surgery type (new or revision) and follow-up period (3 vs 12 months). Missing and incomplete data were excluded from analyses. To compare characteristics by age group and follow-up period, we used the χ^2^ test for categorical variables and the Student *t* test or the Wilcoxon rank-sum test for continuous variables. Significance was 2-tailed, and differences were considered significant at *P* ≤ .05. Analyses were conducted using SAS version 9.4 software (SAS Institute, Cary, NC) and R version 4.1.2 software (R Foundation for Statistical Computing, Vienna, Austria).

## Results

Between 2009 and 2018, 14 hospitals from 7 Canadian provinces conducted prospective surveillance of CSF shunt SSI. Among 269 patients with infection, 53.6% were female and 59.5% were adults. Among shunts with SSI, 106 (90.6%) were ventriculo-peritoneal.

The cumulative CSF shunt SSI rates per 100 procedures from 2009 to 2018 were 3.41 (95% confidence interval [CI], 2.83–4.11) for adults and 3.19 (95% CI, 2.71–3.76) for children, and these rates did not differ significantly. The cumulative CSF shunt SSI rates from 2010 to 2018 were 3.22 (95% CI, 2.68–3.86) for new surgery and 3.47 (95% CI, 2.85–4.23) for revision surgery, and these rates did not differ (Supplementary Fig. 1 online).

Overall, 278 CSF shunt SSI pathogens were identified (Table 1). The most common pathogens included coagulase-negative *staphylococci* (n = 114, 41.0%), followed by *Staphylococcus aureus* (n = 72, 25.9%) and *Cutibacterium* spp (n = 19, 6.8%). Differences in the frequencies of these pathogens were observed between pediatric and adult patient groups (Supplementary Table 2 online).

**Table 1.** Patient Characteristics of Cerebrospinal Fluid (CSF) Shunt SSI Cases by Follow-Up Period, Canada, 2009–2018

Characteristic	Follow-up ≤3 mo (90 d) (n = 221), No. (%)^a^	Follow-Up >3 mo (90 d) (n = 44), No. (%)^a,b^	Total (n = 265), No. (%)^a^	*P* Value
**Age, y**				.850
Mean (SD)	24.0 (25.7)	23.2 (25.7)	23.9 (25.6)	
Median (IQR)	13.8 (0.5–44.0)	14.7 (0.5–35.8)	13.8 (0.5–43.5)	
Range	0.0–84.8	0.0–83.0	0.0–84.8	
**Sex**				.869
Female	118 (53.6)	23 (52.3)	141 (53.4)	
Male	102 (46.4)	21 (47.7)	123 (46.6)	
Median time to infection, d (IQR)	21 (12–40)	156 (119–237)	29 (14–64)	<.0001
**Pathogens (n = 278)** ^c^				.321
**Gram-positive organisms**	200 (86.2)	41 (89.1)	241 (86.7)	
Coagulase-negative *staphylococcus* spp	92 (39.7)	22 (47.8)	114 (41.0)	
*Staphylococcus aureus*	62 (26.7)	10 (21.7)	72 (25.9)	
*Cutibacterium* spp	13 (5.6)	6 (13.0)	19 (6.8)	
*Enterococcus faecalis*	11 (4.7)	0 (0.0)	11 (4.0)	
Other	22 (9.5)	3 (6.5)	25 (9.0)	
**Gram-negative organisms**	25 (10.8)	3 (6.5)	28 (10.1)	
*Pseudomonas aeruginosa*	9 (3.9)	0 (0.0)	9 (3.2)	
*Escherichia coli*	7 (3.0)	1 (2.2)	8 (2.9)	
Other	9 (3.9)	2 (4.3)	11 (4.0)	
**Fungi**	7 (3.0)	2 (4.3)	9 (3.2)	
*Candida* spp	3 (1.3)	0 (0.0)	3 (1.1)	
Other	4 (1.7)	2 (4.3)	6 (2.2)	
**Surgery type**				.653
New placement	122 (56.0)	23 (52.3)	145 (55.3)	
Revision	96 (44.0)	21 (47.7)	117 (44.7)	
**Shunt type**				.585
Ventriculo-peritoneal	192 (88.9)	40 (93.0)	232 (89.6)	
Lumboperitoneal	7 (3.2)	2 (4.7)	9 (3.5)	
Ventriculo-atrial	3 (1.4)	0 (0.0)	3 (1.2)	
Other^d^	14 (6.5)	1 (2.3)	15 (5.8)	

Note. Missing or unknown values were excluded from the analysis. SSI, surgical site infection;

SD, standard deviation; IQR, interquartile range.

a Units unless otherwise specified.

b Infections for the >3-months group includes cases identified between 4 months and 12 months after the surgery.

c In some cases, >1 pathogen per patient were included in the analysis.

d Examples of other shunt types include devices that diverse CSF from a ventricular or subdural space to a distal compartment which can absorb CSF.

The overall median time from shunt surgery to SSI identification was 29 days (interquartile range [IQR], 19–64). Pediatric patients had a shorter median time from shunt surgery to SSI identification (27 days; IQR, 13–48) compared to adult patients (36 days; IQR, 17–74). Among pediatric patients, no difference in demographics or microbiological characteristics were noted when comparing patients aged <1 year to patients aged 1–17 years (Supplementary Table 3 online).

**Fig. 1.**
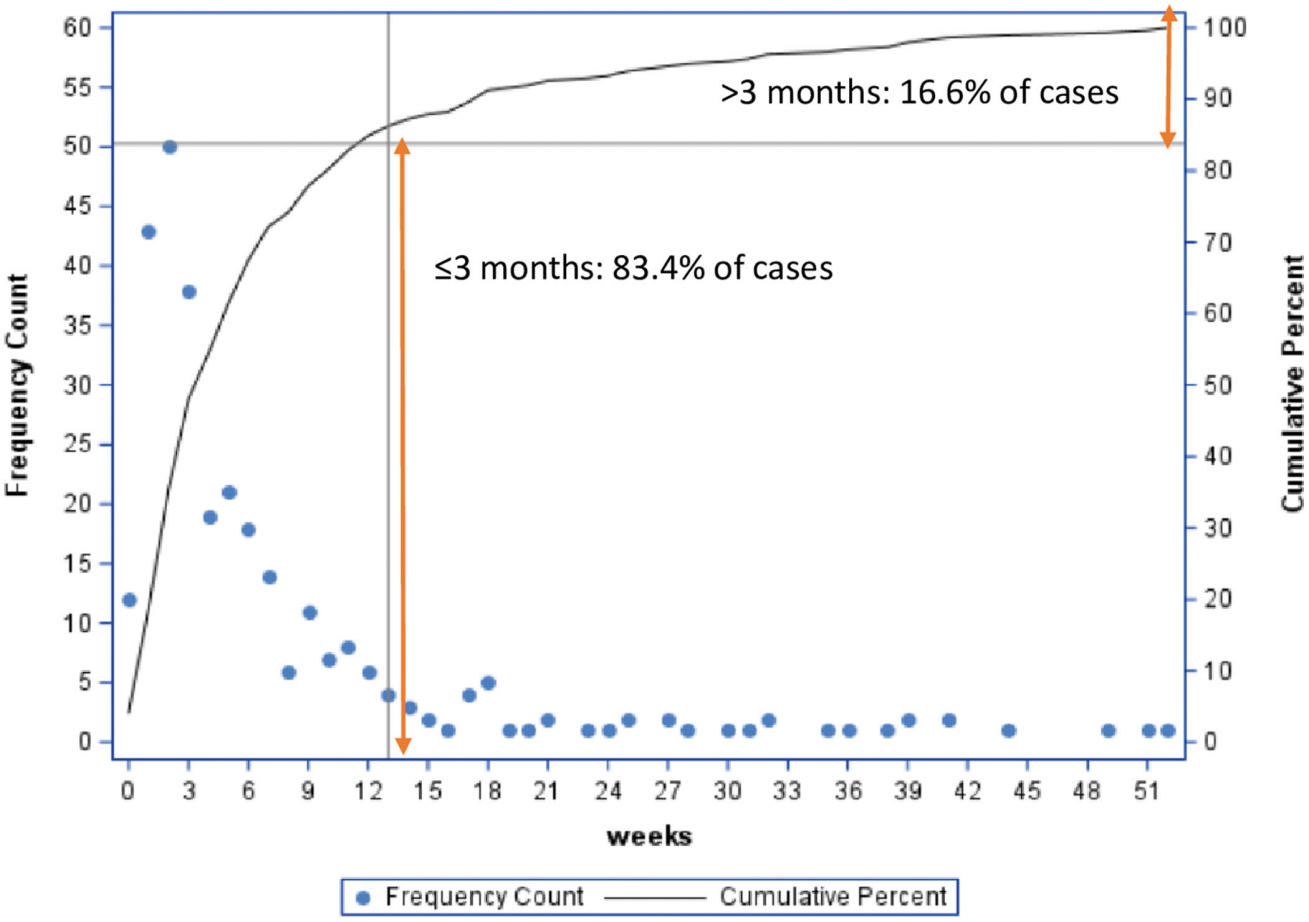
Comparison of CSF shunt associated surgical site infections according to surveillance follow-up of 3 vs. 12 months, Canadian Nosocomial Infection Surveillance Program, 2009-2018.

Between 2009 and 2018, 221 SSIs (83%; 95% CI, 77.5–86.0) were identified within 3 months of the surgery (Fig. 1), and 44 (16.6%) were identified after 3 months (Table 1). The overall SSI rates per 100 procedures at 3 and 12 months were 2.74 (n = 221) and 3.48 (n = 265), respectively. No differences in age distribution, sex, surgery type, shunt type, or infecting organisms were observed when 3- and 12-month periods were compared.

## Discussion

We observed that 83.4% of CSF shunt SSIs occurred within 3 months of surgery, indicating that a shorter follow-up period of 3 months captures most cases. Furthermore, no significant differences in patient characteristics, surgery types, or pathogens were observed when comparing 3-month and 12-month surveillance periods.

The median time from procedure to SSI detection in this study was 29 days, which falls within the proposed 3-month surveillance follow-up period. Our data parallels the experience of other centers. Studies from the United States and Europe in adults and children show that 70%–100% of CSF shunt SSIs are detected within a 90-day window.^7,8^ National Dutch surveillance of implant surgeries found that a 3-month follow-up missed only 6%–14% of SSIs compared to a 12-month surveillance period.^7^

Compared to a 12-month duration of surveillance, a 3-month period for device-associated SSI has several advantages. The shorter follow-up period reduces IPAC resources required to conduct surveillance, freeing them to be used for other high-impact IPAC activities. A shorter surveillance period allows for more timely reporting of infection rates back to healthcare teams, who can make changes to practice where necessary in a more timely manner. Other CNISP surveillance programs have now reduced their postoperative follow-up period for SSI detection to 3 months.^9^

We noted a decrease in the rate of infection associated with shunt placement since our last report, when the overall rate was 4.1 events per 100 procedures compared to 3.2 in this report.^5^ Temporal changes over time could be due to improved application of infection prevention and control measures and an increased awareness over recent decades of patient safety, particularly operating room safety. In addition, grouped measures, or “bundles” of quality improvement interventions have been used successfully to prevent CSF shunt–associated infection.^10^ These bundles include antimicrobial prophylaxis before the first incision, clipping rather shaving of hair, and measures to decrease staff circulation in the operating room and minimizing the time that sterile devices are removed from packaging before insertion.

This study had several limitations. It describes CSF shunt SSIs in a subset of Canadian acute-care hospitals, and these findings may not be generalizable to the general Canadian inpatient population. However, the CNISP network employs standardized collection of detailed data from a large network of sentinel hospitals from multiple provinces over many years.

In conclusion, CSF shunt SSI surveillance for 3 months versus 12 months captures the majority of infections, with no significant differences in patient characteristics, surgery types, or pathogens. A 3-month surveillance period can reduce resources and allow more timely reporting of infection rates and interventions when needed.

## Supplementary material

For supplementary material accompanying this paper visit https://doi.org/10.1017/ice.2022.119. click here to view supplementary material
