# Supplementary material for: Cerebrospinal fluid shunt–associated surgical site infection with three-month versus twelve-month surveillance periods in Canadian hospitals
Source: Infect Control Hosp Epidemiol. 2022 Jun 16;44(6):971–4. doi: 10.1017/ice.2022.119 (PMC10262167; doi:10.1017/ice.2022.119)
Supplement: Supplementary file 1 [file S0899823X22001192sup001.docx]

# **Supplementary**

**Supplementary Table 1**: Surveillance case definitions and eligibility criteria.**Cerebrospinal fluid shunt surgical site infection CNISP surveillance definition:**

Surveillance includes all patients undergoing placement or revision of a cerebrospinal fluid (CSF) shunting device. Those who acquired an infection within 1 year of surgery are included as cases.

**CSF shunt-associated surgical site infection case definition:**

An internalized CSF shunting device is in place **AND** a bacterial or fungal pathogen(s) is identified
from the cerebrospinal fluid **AND** is associated with at least **ONE** of the following:

1. Fever (temperature ≥38º C)
2. Neurological signs or symptoms
3. Abdominal signs or symptoms
4. Signs or symptoms of shunt malfunction or obstruction

**Patient Exclusions:**

•Patients with transcutaneous or external shunting devices or non-shunting devices (e.g. Ommaya reservoir).

•Patients whose CSF was culture positive (bacterial or fungal) at the time of placement of the shunt.

•Infections in which the device associated with the positive organism was not placed at the hospital where the infection was identified, i.e. the hospital should not report the infection.

## **Supplemental Figure 1. a. CSF shunt SSI rates by age within 12 months follow-up period, 2009-2018**


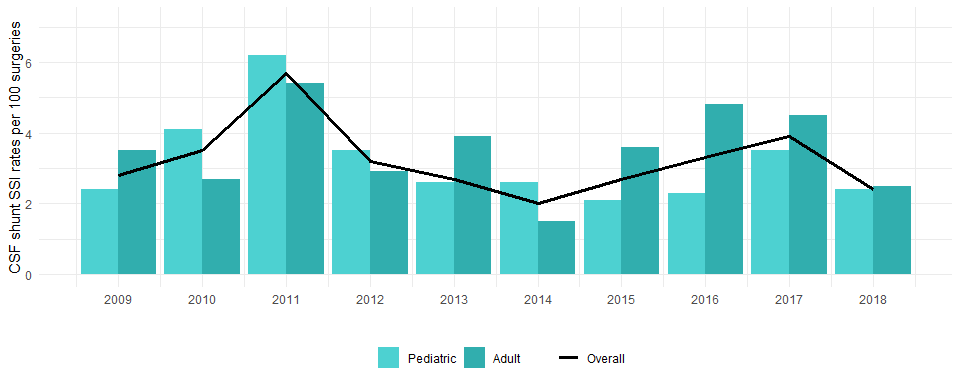


## **Supplemental Figure 1. b. CSF shunt SSI rates by primary vs. revision surgery within 12 months follow-up period, 2009-2018**


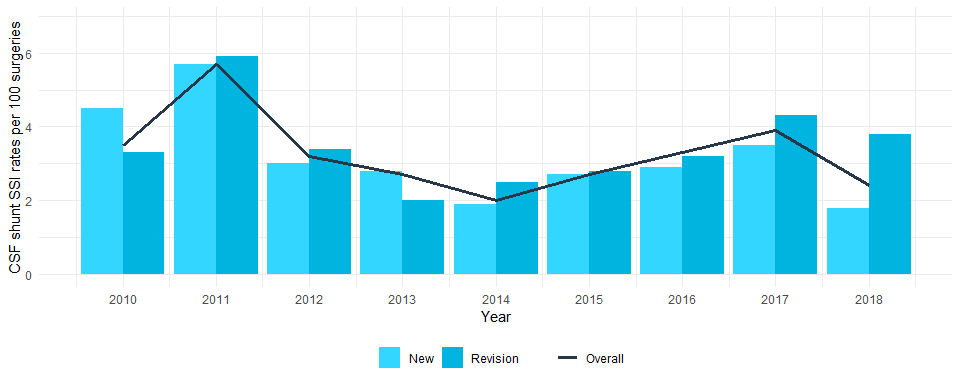


## **Supplemental Table 2. Patient characteristics of CSF shunt SSI cases by age group, Canada, 2009-2018**

|  | **Adult (n=122)** | **Ped (n=147)** | **Total (n=269)** | ***p*-value** |
| --- | --- | --- | --- | --- |
|  |  |  |  |  |
| **Age (years)** |  |  |  |  |
| Mean (SD) | 47.5 (18.9) | 3.8 (5.3) | 23.9 (25.6) |  |
| Median (IQR) | 45.7 (29.9, 64.1) | 0.6 (0.2, 6.8) | 13.8 (0.5, 43.3) |  |
| Min – Max | 18.9 - 84.8 | 0.0 - 17.9 | 0.0 - 84.8 |  |
| **Sex** |  |  |  | 0.077 |
| Female | 72 (59.5%) | 70 (48.6%) | 142 (53.6%) |  |
| Male | 49 (40.5%) | 74 (51.4%) | 123 (46.4%) |  |
| **Median Surgery to onset date (days), (IQR)** | 36 (17, 74) | 27 (13, 48) | 29 (14, 64) | 0.035 |
| **Pathogens (n=279)^a^** |  |  |  | 0.013 |
| **Gram positive** | 115 (90.6%) | 127 (83.6%) | 242 (86.7%) |  |
| Coagulase-Negative *Staphylococcus* sp. | 52 (40.9%) | 62 (40.8%) | 114 (40.9%) |  |
| *Staphylococcus aureus* | 31 (24.4%) | 41 (27.0%) | 72 (25.8%) |  |
| *Cutibacterium* species | 15 (11.8%) | 5 (3.3%) | 20 (7.2%) |  |
| *Enterococcus faecalis* | 6 (4.7%) | 5 (3.3%) | 11 (4.7%) |  |
| Other gram positives | 11 (8.7%) | 14 (9.2%) | 25 (9.0%) |  |
| **Gram negative** | 7 (5.5%) | 21 (13.8%) | 28 (10.0%) |  |
| *Pseudomonas aeruginosa* | 4 (3.1%) | 5 (3.3%) | 9 (3.2%) |  |
| *Escherichia coli* | 3 (2.4%) | 5 (3.3%) | 8 (2.9%) |  |
| Other Gram negatives | 0 (0.0%) | 11 (7.2%) | 11 (3.9%) |  |
| **Fungi** | 5 (3.9%) | 4 (2.6%) | 9 (3.2%) |  |
| *Candida* species | 3 (2.4%) | 3 (2.0%) | 6 (2.2%) |  |
| Other Fungi | 2 (1.5%) | 1 (0.7%) | 3 (1.1%) |  |
| **Surgery type** |  |  |  | 0.155 |
| New placement | 61 (50.4%) | 84 (59.2%) | 145 (55.1%) |  |
| Revision | 60 (49.6%) | 58 (40.8%) | 118 (44.9%) |  |
| **Shunt type** |  |  |  | < 0.001 |
| Ventriculo-peritoneal | 106 (90.6%) | 126 (88.7%) | 232 (89.6%) |  |
| Lumbo-peritoneal | 8 (6.8%) | 1 (0.7%) | 9 (3.5%) |  |
| Ventriculo-atrial | 2 (1.7%) | 1 (0.7%) | 3 (1.2%) |  |
| Other | 1 (0.9%) | 14 (9.9%) | 15 (5.8%) |  |

Note: Missing or unknown values were excluded from the analysis.

Abbreviations: ped, pediatric; SD, standard deviation; IQR, interquartile range

a. In some cases, more than one pathogen per patient was included in the analysis

## **Supplemental Table 3. Patient characteristics of CSF shunt SSI cases for pediatric patients by age group, Canada, 2009-2018**

|  | **Enfants (<one year of age)**  **(n=79)** | **Other pediatric patients (1<=year of age<18)**  **(n=65)** | **Total (n=144)** | ***p*-value** |
| --- | --- | --- | --- | --- |
|  |  |  |  |  |
| **Age (years)** |  |  |  |  |
| Mean (SD) | 0.3 (0.2) | 8.1 (5.2) | 3.8 (5.3) |  |
| Median (IQR) | 0.2 (0.1, 0.4) | 8.3 (3.1, 13.1) | 0.6 (0.2, 6.8) |  |
| Min – Max | 0.0 – 0.9 | 1.00 - 17.9 | 0.0 – 17.9 |  |
| **Sex** |  |  |  | 0.254 |
| Female | 35 (44.3%) | 35 (53.9%) | 70 (48.6%) |  |
| Male | 44 (55.7%) | 30 (46.1%) | 74 (51.4%) |  |
| **Median Surgery to onset date (days), (IQR)** | 23 (12, 49) | 27 (14, 46) | 27 (13, 48) | 0.779 |
| **Pathogens (n=152)^a^** |  |  |  | 0.114 |
| **Gram positive** | 65 (82.3%) | 62 (84.9%) | 127 (83.6%) |  |
| Coagulase-Negative *Staphylococcus* sp. | 37 (46.8%) | 25 (34.3%) | 62 (40.8%) |  |
| *Staphylococcus aureus* | 15 (19.0%) | 26 (35.6%) | 41 (27.0%) |  |
| *Cutibacterium* species | 1 (1.3%) | 4 (5.5%) | 5 (3.3%) |  |
| *Enterococcus faecalis* | 6 (7.6%) | 1 (1.4%) | 7 (4.6%) |  |
| Other gram positives | 6 (7.6%) | 6 (8.2%) | 12 (7.9%) |  |
| **Gram negative** | 12 (15.2%) | 9 (12.3%) | 21 (13.8%) |  |
| *Pseudomonas aeruginosa* | 2 (2.5%) | 3 (4.1%) | 5 (3.3%) |  |
| *Escherichia coli* | 3 (3.8%) | 2 (2.7%) | 5 (3.3%) |  |
| Other Gram negatives | 7 (8.9%) | 4 (5.5%) | 11 (7.2%) |  |
| **Fungi** | 2 (2.5%) | 2 (2.8%) | 4 (2.6%) |  |
| *Candida* species | 2 (2.5%) | 1 (1.4%) | 3 (2.0%) |  |
| Other Fungi | 0 | 1 (1.4%) | 1 (0.6%) |  |

Note: Missing or unknown values were excluded from the analysis.

Abbreviations: ped, pediatric; SD, standard deviation; IQR, interquartile range

a. In some cases, more than one pathogen per patient was included in the analysis
